# End-of-Life in Oncologic Patients’ Dream Content

**DOI:** 10.3390/brainsci10080505

**Published:** 2020-08-01

**Authors:** Alessandro Cicolin, Michele Boffano, Guglielmo Beccuti, Raimondo Piana, Alessandra Giordano

**Affiliations:** 1 Sleep Medicine Center, Department of Neuroscience, University of Torino, 10126 Torino, Italy; alessandra.giordano@unito.it; 2 Orthopaedic Oncologic Surgery, Department Oncological and Reconstructive Orthopedics, University of Torino, 10126 Torino, Italy; mboffano@cittadellasalute.to.it (M.B.); rpiana@cittadellasalute.to.it (R.P.); 3 Division of Endocrinology, Diabetes, and Metabolism, Department of Medical Sciences, AOU Città della Salute e della Scienza, University of Torino, 10126 Torino, Italy; guglielmo.beccuti@unito.it

**Keywords:** end of life, oncology, dream content, sleep, memory, memory consolidation, health communication

## Abstract

Both non-rapid eye movements and rapid eye movements sleep facilitate the strengthening of newly encoded memory traces, and dream content reflects this process. Numerous studies evaluated the impact of diseases on dream content, with particular reference to cancer, and reported the presence of issues related to death, negative emotions, pain and illness. This study investigates death and illness experiences in 13 consecutive patients with sarcoma compared to paired controls, early after diagnosis, evaluating dream contents, fear of death, mood and anxiety, distress, and severity of disease perception (perceived and communicated). Ten patients and 10 controls completed the study. Dream contents were significantly different between patients and normative data (DreamSat) and patients and controls (higher presence of negative emotions, low familiar settings and characters and no success involving the dreamer). Illness and death were present in 57% of patients’ dreams (0% among controls), but no differences emerged between patients and controls in regard to anxiety and depression, distress and fear of death, even if the severity of illness was correctly perceived. The appearance of emotional elements in dreams and the absence of conscious verbalization of distress and/or depressive or anxious symptoms by patients could be ascribed to the time required for mnestic elaboration (construction/elaboration phase) during sleep.

## 1. Introduction

Memories are a combination of specific traces encoded at the time of an event, along with knowledge, expectations, beliefs and experiences of such event. According to Frederic Bertlett [1], the memory process is guided by schemata, or general organizing structures, a framework for the organization and understanding of information that aid in encoding and retrieval. Schemata capture the ability to systematically organize a multitude of facts and experiences and to extract the general idea which enables the transfer of pre-existing knowledge to novel stimuli and situations [2].

Sleep processes are necessary for optimal declarative and non-declarative memory consolidation and memory stabilization [3]. Sleep, indeed, facilitates the abstraction of rules (schemata formation), the integration of knowledge into existing schemata (schemata integration) and creativity that requires the disbandment of existing patterns (schemata disintegration). Slow wave sleep (SWS) is supposed to particularly enhance declarative memories, whereas rapid eye movements (REM) sleep preferentially supports procedural and emotional memory aspects. From this perspective, we can imagine dreaming as a “background noise” associated with all of these processes.

Regarding memory consolidation, it should be noted that it is not revealed under all circumstances but is linked to specific psychological conditions. Our unconscious elaboration is not neutral but connected to our emotional experiences. Indeed, emotions have been shown to modulate the sleep-related reorganization of memories, and neuroimaging data provide neurobiological support for an implication of sleep and dreaming in some important functions, such as emotional regulation [4]. REM sleep has a well-known role in the processing of emotional waking-life experiences supporting emotional memory consolidation. This is probably because the neuroanatomical regions implicated in emotional processes during wakefulness are also responsible for the neurophysiological background of REM sleep [5]. Despite the theory that suggests that dreams are epiphenomena of sleep without any natural function, the evolutionary hypothesis of Revonsuo suggests that dreams could support a behavioral advantage by selecting and simulating threatening waking events in order to maintain threat-avoidance skills [6]. Neuroimaging studies on regional brain activity during sleep can explain specific dreams’ features and can help to support this theory, and the study of changes in brain activity and mental content across all sleep-wake states could provide neurobiological support for an implication of sleep and dreaming in important functions such as emotional regulation [4].

Regarding memory processes, on the opposite side of remembering, forgetting may be the result of interference or decay, but it is also possible that the brain “tags” information for preferential processing during sleep, enhancing some memories while allowing others to fade [7]. Recent research suggests that information is tagged based on its importance to the individual, with this information undergoing selective reactivation [8]. Emotional material is relevant to an individual because of its inherent value for future survival. Consistently, ample evidence suggests that the brain promotes memories for both threatening and appetitive stimuli [9,10,11]. Such information is remembered better than neutral information when consolidated across wakefulness, but this effect is further enhanced by sleep [11,12]. There may also be a role for reactivation and reorganization specifically during REM sleep, as many studies have observed an association between emotional memory and the amount of REM sleep obtained [10,12,13]. Translational evidence seems to converge that sleep, and in particular REM sleep, is involved in the consolidation of fear- and safety-relevant information [14]. More specifically, it seems that REM sleep may serve the purpose of emotional valence re-evaluation and adjustment [15].

The link between physical, mental, and emotional conditions, health, and dream content has always been a topic of interest, not only in the academic–scientific environment. One of the most interesting questions concerns the possible role and usefulness of the analysis of dream content in the evaluation of the elaboration and perception of illness, discomfort, psychological and emotional reaction, and of general psycho-physical well-being.

Since 1953, when Aserinsky and Kleitman [16] discovered REM sleep, oneiric activity has been systematically studied in sleep laboratories and sleep research demonstrated that people mostly dream during REM sleep (80%) and, less frequently (6.9%) and with different features, during non-rapid eye movements (NREM) sleep [17,18,19,20,21]. Even if recent studies have shown that REM sleep can occur without dreams and oneiric activity without REM sleep, REM sleep and dreams are frequently associated [22,23].

In the literature, numerous studies evaluated the impact of diseases on dream content, with particular reference to cancer [20,24,25,26,27,28,29]. The above-mentioned studies highlighted that the dream content of patients treated for cancer differed from control subjects, by presenting a greater presence of issues related to death, conflicting situations, temporary denial of the disease, nightmares, negative emotions, medical figures, anatomical images and topics such as pain and illness. These results seem to be consistent with the continuity hypothesis expressed by Domhoff [30], according to which concerns found in dreams are the same as in the dreamer’s daily life [31] and they also agree with Hobson’s theory [32] that considers dream content as transparent, therefore readable through direct access that does not require decoding, thus reflects the dreamer’s vision of the world.

Events and memories from waking life are frequently incorporated into dreams, either as classical day-residues in the following night or after a “dream lag” of about 5–7 days [33,34,35]. Particularly engaging learning experiences have a more robust influence on dream content when compared to more passive experiences, which might lead to underestimations of experience-related dream incorporations [36].

We hypothesized that the complex emotional reaction to oncological diagnosis can be present in the oneiric content through the elements that recall concerns and anxieties related to the outcome of illness and to death.

This study aimed to investigate death and illness experiences in patients affected by sarcoma (rare malignancies of mesodermal origin with reduced prognosis due to their aggressive biology) through a dream content analysis. We also decided to investigate the impact of the diagnosis on mood (levels of stress, anxiety and depression-like symptoms), and patients’ awareness of their clinical condition, intended as the severity of perceived illness during the communication of the diagnosis by physicians, to assess any discrepancies between “perception” and “intention” regarding diagnosis communication.

## 2. Materials and Methods

For eight months, all consecutive outpatients referred to the Department of Oncological and Reconstructive Orthopedics in the University Hospital “Città della Salute e della Scienza di Torino”, Italy, were evaluated. Inclusion criteria were the following: age between 25 and 70 years, primary sarcoma diagnosis, eligibility for chemotherapy and/or surgery (not palliative care). We also evaluated healthy volunteers paired by gender, age and education. For both patients and controls exclusion criteria were: actual (except for patients) cancer diagnosis, having received previous cancer or other potentially lethal disease diagnoses, being related to oncologic patients, impaired cognitive ability (MMSE > 24), severe or untreated psychopathology, following the Diagnostic and Statistical Manual of Mental Disorders, Fifth Edition [37], neurological disorders, dementia, immunologic conditions or diseases, sleep disorders, following the International Classification of Sleep Disorders III edition [38], use of antidepressants, benzodiazepines and hypnotic drugs. The Local Ethical Committee approved the protocol, and patients and volunteers signed the informed consent form. The study was conducted in accordance with the principles of the Declaration of Helsinki [5] following the Good Clinical Practices.

Patient and volunteer evaluation included the following: (a) demographic and clinical data; (b) a sleep and dream diary in which subjects were asked to describe their sleep quality and duration and their dreams over one week, if present (within 7–15 days after diagnosis to potentially detect early signs of the impact of diagnosis); (c) The Collett–Lester fear of death [39], a self-assessment scale that investigates the levels of anxiety and concern about death; (d) the Hospital Anxiety and Depression scale (HADS) [40]; (e) The Distress Thermometer, a single-item tool using a 0 (no distress) to 10 (extreme distress) point Likert scale resembling a thermometer. Patients rated their level of distress over the past week [41,42,43]. Patients were also asked to fill a visual analog scale (VAS) indicating a position along a continuous line between two end-points (0–10 cm), assessing perception of the severity of their illness; in a similar VAS, the doctor assessed the global severity of illness as communicated to the patient. The reported dream contents were coded according to the Hall and Van de Castle coding system [44]. Dreams were blindly scored by a sleep medicine board-certified psychologist; difficult or ambiguous issues were resolved in group discussions. The alphanumeric codes were uploaded into a DreamSAT spreadsheet, (free web version), which provided frequencies for the total series of dream reports and percentage calculations for patients compared to controls [45,46]. Coded dream data were also statistically compared to normative data using Adam Schneider’s DreamSAT [47].

Data were analyzed by IBM SPSS 25.0 for Windows [48] using paired Τ-test to compare the mean score obtained from the Collet–Lester Scale, the HADS and distress thermometer between patients and volunteers, unpaired T-test to compare VAS-communicated and -perceived severity of illness, and the chi-square test (χ^2^) on the presence/absence of illness in dream content between patients and healthy volunteers. Dream contents were analyzed using DreamSat tools. Results are presented in terms of mean ± SD with significance levels at *p* < 0.05 (*) and *p* < 0.01 (**).

## 3. Results

Out of 21 eligible outpatients, 8 refused to take part in the study, and 3 dropped out (they did not return the sleep diary). Ten patients completed the study (3/7 m/f; mean age: 47.80 ± 14.19 years; education: 11.30 ± 4.47 years; total number of reported dreams: 23; diagnosis: osteosarcoma (6), soft tissue sarcoma (4); suggested therapy: surgery (9), chemotherapy (1); average total sleep time (TST): 404.18 ± 102.06 min; average sleep efficiency (SE): 81.59 ± 17.7% ) paired with 10 healthy volunteers (3/7 m/f; mean age: 45.30 ± 12.85 years; education: 14.70 ± 2.98 years; total number of reported dreams: 15; average TST: 464.7 ± 46.63 min; average SE: 93.10 ± 6.44%). TST and SE are subjective measures obtained from subjects’ sleep and dream diaries. No statistically significant differences emerged in gender, age and education of oncological patients when compared to healthy volunteers.

Regarding self-assessment scales administered to the subjects of both samples, that is, the HADS, the Distress Thermometer and the Collett–Lester scale, no scores were above the cut-off. Furthermore, no statistically significant differences emerged between oncology patients and healthy volunteers in self-assessment scales (see Table 1).

### Dream Content Analysis

There were no oneiric reports from male subjects belonging to both groups over seven nights of registration, thus, the relationship between gender and the total number of reported dreams was significant. Only the results of female (7/10) patients and volunteers are reported.

A statistically significant difference has emerged between volunteers and patients regarding the presence of illness in the dream content: among healthy volunteers there were no dreams related to illness and death (patients: 4/7 (57%); controls 0/7 (0%); *p* = 0.005).

Patients who dreamt of illness reported a greater number of dreams compared to the subjects who did not (dreams including illness: 20; dreams with no illness: 3; *p* = 0.059).

Table 2 describes the dream recall frequency per night over 1 week of dream recording for both patients and volunteers.

DreamSat categories showed statistically significant differences between patients and normative data, as well as between healthy volunteers and cancer patients.

Table 3 shows DreamSat categories and output.

Table 4 shows significant results regarding the comparison between patients and healthy volunteers. Patients had significantly fewer animal characters, fewer friendly interactions and fewer successes involving the dreamer. No statistically differences resulted in regard to other DreamsSat categories.

In Table 5, the significant data that emerged from the comparison between patients and normative data are reported. The presence of negative emotions was higher in the patient sample, which also had a lower percentage of known characters or animals, less friendly interactions or physical aggressions, a lower presence of familiar settings and no success involving the dreamer.

## 4. Discussion

The research hypothesis was to investigate the incorporation of the “end-of-life” concept in the emotional experiences of oncologic patients. Our results surprisingly highlighted that the level of anxiety, depression, distress and the fear of death was not above the questionnaires cut-off in both patients and healthy volunteers, and no statistically significant differences emerged between the two groups, even if the severity of diagnosis was correctly perceived by patients. Regarding dream content, we found no dreams related to illness and death among the controls, and a relevantly higher number of dreams was registered among patients who described illness in their dreams. Despite the fact that the sample size reduced the power of statistical analysis, the results almost reached statistical significance. Patients had significantly fewer friendly interactions and successes involving the dreamer when compared to volunteers. Besides, patients showed a higher presence of negative emotions, very low familiar settings and characters and no success involving the dreamer compared to female normative data. Moreover, no dreams were reported by both male patients and controls.

Our results further confirm the continuity hypothesis expressed by Domhoff [30] and data from the literature [49,50], according to which the thoughts and concerns in dream reports are the same as those that appear in the dreamer’s waking life, and with the studies of De Cicco and colleagues and Giordano and colleagues [26,51] that have highlighted, in cancer patients, a greater presence of nightmares, negative images and emotions, experiences and images related to illness, death, hospitalization, and pain. More than half of the women in our sample reported explicit references to their illness, hospital settings, and death. These reports are absent among the dreams of healthy volunteers. Furthermore, patients, when compared with healthy female volunteers, showed a lower presence of friendly interactions and successes involving the dreamer, placing the emphasis on the dislike of self, emerging from the total absence of positive outcomes following a personal and direct effort. The data also showed that patients who reported illness in their dream diary were also those who dreamed the most. The particularly emotional dream content remained in the memory because it strongly interconnected with the traumatic experience when awake, indeed, dream incorporations have been suggested to reflect processes of memory consolidation. The appearance of newly encoded information in our daydreams, thoughts, and dreams may be an observation of predominant importance in understanding the activities of the mind and brain during both wake and sleep [52,53,54].

Regarding the elaboration of the concept of death, it can emerge in a substantially conscious way during clinical interviews through verbalization, can be quantified through specific evaluation scales and can be unconsciously reflected in the oneiric content.

Although subjects were facing a very delicate phase of their diagnostic–therapeutic path, they did not show significant anxiety or depression symptoms, and it could not be due to unawareness of diagnosis since a strong concordance emerged between the severity of disease communicated by doctors to patients and the severity of illness perceived by patients themselves. This phenomenon could rely on the behavioral response of subjects, who experienced trouble in verbalizing their emotions, or it could be expected as an effective early coping strategy. Nevertheless, it could be also speculated that patients found themselves in a sort of “mnestic construction/elaboration phase” required for schemata formation and integration, meaning that health issues were not yet cognitively elaborated due to absent or insufficient mnestic traces.

Given the frequency of depression and anxiety symptoms in oncologic patients [55,56,57], the absence of differences between healthy volunteers and patients for what concerns the level of anxiety, depression and distress is intriguing. Each patient, with their psychological and social characteristics, has a peculiar history and specific tools to manage difficulties. These very unique characteristics can strongly influence the emotional response to traumatic events such as cancer diagnosis, whether in a positive (functional coping mechanisms) or in a negative way (difficult or no adaptation to the disease). It is unlikely that the comparable levels of anxiety and depression, emotional distress, and fear of death between patients and healthy subjects could be due to successful coping strategies implemented by patients in early stages after diagnosis (2 weeks). As previously stated, both REM and NREM sleep are necessary to create the mnesic engram and reorganize emotional memory, which are essential for mental health and well-being. Specifically, emotions seem to have an important modulating effect in the course of memory reorganization, which remains to be further characterized. Sleep modulates the processes responsible for qualitative changes in memory [2]. Until REM and NREM sleep processes (and the associated dream activity) cease to create the mnestic trace, the coping strategies to manage events and related emotions can be impossible or incomplete. As a result, subjects can perfectly understand and be aware of their conditions, but they do not (yet) develop anxiety, depression or death-related fears. The apparent absence of psychological distress in these patients in the first days after diagnosis communication could be ascribed to sleep (REM-NREM)-related cognitive processes and a not-yet completed schemata formation/integration, given that the process may require some nights to be fully completed. If it is supposed that dreams are linked to the evolution of memories and memory processes during sleep are relevant to the managing of emotional responses, then the targeted manipulation of sleep and a memory reorganization might help in managing mental disorders and dysfunctional emotional responses [2].

One of the limitations of this research is certainly the small number of subjects. This was due to multiple causes: the difficulty in finding patients with no other comorbidities and the rare frequency of sarcoma; the exclusion of subjects with previous cancer diseases and those with cognitive impairment or obvious language barriers. Last but not least, many subjects refused to participate in such a critical period of their life.

Moreover, patients were required to send back by mail the sleep and dream diary after 7 nights of recordings. In some cases, patients did not return the diary or it was delivered too late for the analysis purpose. In our opinion, the main reason why subjects did not return the diary was the difficult moment they were experiencing—filling a sleep diary would not have been of high priority. In addition, the research did not consider any re-testing, thus removing the possibility to follow up any changes in the dream content and/or in psychological variables (distress, mood and anxiety). To better understand the timing of possible changes in the emotional response (questionnaires) and in the dream content (dream diary), further studies should include a follow up and re-testing, at least at 3, 6, and 12 months after diagnosis. Furthermore, adding polysomnographic (PSG) measures could help to better understand the neural bases of dreaming and to correlate sleep quality and dream recall rate.

Finally, we obtained no dream recall from males, both patients and volunteers. In the literature, women reported a higher frequency of dream recall than men and dream recall is described to progressively decrease with age in the male population [58,59]. Our data could be due to this phenomenon, amplified by the small sample. Alternatively, this result may not reflect the inability to recall, but rather low compliance to the study or a lesser inclination of the male gender to talk about itself.

## 5. Conclusions

The appearance of the emotional elements in dreams and the absence of conscious verbalization of depressive or anxious symptoms by patients probably represents the main and novel finding of the study and it could be ascribed to the time required for mnestic elaboration (construction/elaboration phase). Given that elaboration of coping strategies and both early and long-term emotional reactions are influenced by all (emotional and cognitive) experiences associated with an event, it is evident that communication of the diagnosis can deeply modify the illness experience. If a clear diagnosis, a proper treatment project and psychological support are offered while the mnesic trace is being formed, it will not avoid distress but hopefully will reduce the risk of depression [60]. In order to better detect possible changes in the emotional response (e.g., depressive episodes) and in dream content, further studies should include an extended follow up. Moreover, performing PSGs could help to better understand the neural bases of dreaming.

## Figures and Tables

**Table 1:** Distress, fear of death, anxiety and depression in patients and volunteers.

	Patients	Controls	*p*
**Distress Thermometer**	4.65 ± 3.0	4.70 ± 1.7	n.s.
**Collet–Lester (fear of death)**			
your death	18.38 ± 6.0	20.40 ± 5.4	n.s.
dying	27.75 ± 8.5	26.20 ± 8.0	n.s.
death of others	25.88 ± 6.6	25.80 ± 4.6	n.s.
other people dying	27.75 ± 6.3	29.80 ± 6.4	n.s.
**HADS**			
anxiety	9.00 ± 1.2	8.90 ± 1.4	n.s.
depression	12.50 ± 1.8	12.70 ± 1.8	n.s.

n.s. = not significant. No significant differences were also obtained between doctors’ and patients’ visual analog scale (VAS) (4.50 ± 2.01 vs. 4.55 ± 2.19 cm, *p* = n.s.).

**Table 2:** Dream recall frequency per night.

	Night 1	Night 2	Night 3	Night 4	Night 5	Night 6	Night 7	Tot N Dreams
**Patients**	2	5	4	4	2	3	3	23
**Volunteers**	3	3	3	0	2	1	3	15

**Table 3:** DreamSat categories.

**Characters**
Male/Female Percent
Familiarity Percent
Friends Percent
Family Percent
Dead and Imaginary Percent
Animal Percent
**Social Interaction Percents**
Aggression/Friendliness Percent
Befriender Percent
Aggressor Percent
Physical Aggression Percent
**Settings**
Indoor Setting Percent
Familiar Setting Percent
**Self-Concept Percents**
Self-Negativity Percent
Bodily Misfortunes Percent
Negative Emotions Percent
Dreamer-Involved Success Percent
Torso/Anatomy Percent
**Dreams with at Least One:**
Aggression
Friendliness
Sexuality
Misfortune
Good Fortune
Success
Failure
Striving

**Table 4:** Oneiric content in female patients vs. healthy volunteers.

	Female Patients	Female Controls	*p*
**Characters**			
Animals	0%	6%	* 0.033
**Social Interactions**			
Friendly character	0%	67%	** 0.007
**Self-Concept**			
Dreamer-Involved Success	0%	50%	* 0.04

*p* < 0.05 (*) and *p* < 0.01 (**).

**Table 5:** Oneiric content in female patients vs. normative data.

	Female Patients	Normative Data	*p*
**Characters**			
Familiarity	38%	58%	* 0.028
Animals	0%	4%	* 0.027
**Social Interactions**			
Friendly character	0%	47%	** 0.000
Physical aggression	0%	34%	** 0.003
**Setting**			
Familiar	17%	79%	** 0.001
**Self-Concept**			
Negative emotions	100%	80%	* 0.010
Success involving the dreamer	0%	42%	* 0.016
**Dreams with at Least One:**			
Success	0%	8%	* 0.033

*p* < 0.05 (*) and *p* < 0.01 (**).
